# Pediatricians’ and health visitors’ views towards detection and management of maternal depression in the context of a weak primary health care system: a qualitative study

**DOI:** 10.1186/1471-244X-14-108

**Published:** 2014-04-11

**Authors:** Eirini Agapidaki, Kyriakos Souliotis, Suzanne F Jackson, Vassiliki Benetou, Stylianos Christogiorgos, Christina Dimitrakaki, Yannis Tountas

**Affiliations:** 1 Centre for Health Services Research, Department of Hygiene, Epidemiology and Medical Statistics, University of Athens Medical School, 115 27 Athens, Greece; 2 Faculty of Social Sciences, University of Peloponnese, Korinth, Greece; 3 Dalla Lana School of Public Health, University of Toronto, Toronto, Canada; 4 Department of Hygiene, Epidemiology and Medical Statistics, University of Athens Medical School, Athens, Greece; 5 Department of Child Psychiatry, University of Athens Medical School, “Aghia Sophia” Children’s Hospital, Athens, Greece

**Keywords:** Maternal depression, Pediatricians, Health visitors, Primary healthcare, Mental healthcare, Greece, Europe

## Abstract

**Background:**

The present study’s aim has been to investigate, identify and interpret the views of pediatric primary healthcare providers on the recognition and management of maternal depression in the context of a weak primary healthcare system.

**Methods:**

Twenty six pediatricians and health visitors were selected by using purposive sampling. Face to face in-depth interviews of approximately 45 minutes duration were conducted. The data were analyzed by using the framework analysis approach which includes five main steps: familiarization, identifying a thematic framework, indexing, charting, mapping and interpretation.

**Results:**

Fear of stigmatization came across as a key barrier for detection and management of maternal depression. Pediatric primary health care providers linked their hesitation to start a conversation about depression with stigma. They highlighted that mothers were not receptive to discussing depression and accepting a referral. It was also revealed that the fragmented primary health care system and the lack of collaboration between health and mental health services have resulted in an unfavorable situation towards maternal mental health.

**Conclusions:**

Even though pediatricians and health visitors are aware about maternal depression and the importance of maternal mental health, however they fail to implement detection and management practices successfully. The inefficiently decentralized psychiatric services but also stigmatization and misconceptions about maternal depression have impeded the integration of maternal mental health into primary care and prevent pediatric primary health care providers from implementing detection and management practices.

## Background

Maternal depression is a common mood disorder that occurs in women especially during child-bearing years with prevalence rates ranging from 10-20% [1,2]. Hormonal, psychological and social factors in the prenatal and postnatal period as well as in early child rearing years have been strongly linked to the onset of depression [3]. Maternal depression affects child’s physical, mental and social health [4-6], but also cognitive, and socio-emotional development [7,8]. It has an indirect impact in the use of pediatric health care services and pediatric preventive practices. Underuse of child health preventive practices and overuse of pediatric emergency services have been found in various samples of depressed mothers [9-12].

Pediatric primary health care professionals (e.g. pediatricians and health visitors) usually have the most frequent contact with women of childbearing age and therefore they are in an advantaged position to detect maternal depression during the well-child visits [13-15]. Even though most pediatric primary health care professionals acknowledge the importance of prevention of maternal depression, in fact they fail to contribute effectively to prevent it [16]. In many cases severe depression is detected in the context of primary health care but mild or moderate depression is seldom identified [17].

The early detection and management of maternal depression is recommended by the European Union policy framework which mentions for parenting and the early years of life’ as a major action area [18].

Both the high prevalence of mental disorders in the community and the reform of mental health services (e.g. reduction of psychiatric hospital beds) have resulted in a larger burden of people facing mental health problems in primary care settings [19]. Despite this however, the detection and management of mental disorders in primary care is still understudied.

Research suggests that the quality and effectiveness of a primary health care system affects the recognition and management of mental disorders [19-21].

Hence, in order to better understand the role of pediatric primary health providers in detection and management of maternal depression, it is imperative to investigate this role within the context of the quality and effectiveness of the primary health care system as such.

Previous studies in Europe and internationally determined the strength of a primary healthcare system according to its performance in relation to: a) primary care structure (governance, economic conditions of primary care system, workforce development) and b) the services delivery process (access to PC services, comprehensiveness of PC services, continuity of care, coordination of care) [22,23]. For example the primary health care systems in European Union countries fall into three categories: weak (e.g. Austria, Iceland, Ireland, Greece), medium (e.g. Norway, Sweden, France) and strong (e.g. United Kingdom, the Netherlands, Denmark), in terms of quality, equity and costs of care [22]. The main aspects of a strong primary care system are: universal access, better coordination and continuity of care, team-based provision of services, higher quality of care, focus on prevention and early detection and management of physical and mental diseases [24]. Strong primary health care systems lead to better population health outcomes, reduction in avoidable hospitalizations and unnecessary specialist care but also to better and more equitable access to health services [25].

Specifically in Greece the health care system comprises a combination of public, public contract and public reimbursement models and is based on public-private partnerships [26]. Within this system primary care is provided by: a) the rural primary health centers and the outpatient clinics of public hospitals, both incorporated to the public national health system, b) the health services affiliated to and financed by the social insurance funds, c) the services and individual providers (solo physician setting) of the private sector who have contractual agreements with the social insurance funds [26]. Patients can also have access to private health services and providers by paying out of pocket on a fee for service basis [27]. Public primary care centers are staffed with specialists (e.g., specialist of internal medicine, pediatrician, gynecologist, dentist, radiologists, microbiologists, nurses, midwives and social workers). Each primary health center caters for 10,000 – 30,000 citizens. The primary care system in Greece is hospital-oriented, focused on the provision of curative services rather than disease prevention, health promotion and home-care services [28]. There is no gatekeeping system (e.g. referrals to mental health or other specialists), while continuity of care between primary and secondary health services is weak, if non-existent [26].

Pediatric primary care is provided by both the public and private sectors. Public pediatric primary care is provided by the pediatric outpatient clinics of the public hospitals and the public pediatric primary health centers. Pediatric primary care centers (called “Centers for Maternal and Child Protection”) are part of the public primary settings and staffed by pediatricians, nurses and health visitors. Pediatric primary care is also provided by the private sector through contractual agreements with the social insurance funds. Patients do not need to self-fund access since the majority of the private pediatric settings have contractual agreements with social insurance funds.

The majority of private pediatric primary settings are medical practices run by a single pediatrician-solo practitioner (solo practice). Since the public pediatric primary care centers are few and located in urban areas, the care in semi-urban and rural areas is provided mainly by private pediatric practices. The available services for referral in cases of mothers with depression are: the psychiatric hospitals, psychiatric outpatient clinics of general public hospitals, community mental health centers (which are scarce and mainly clustered in the large urban areas) and private mental health specialists (e.g., psychiatrists) [26].

In an effort to better understand the context of pediatric primary health care in connection with the detection and management of maternal depression, studies in countries with strong primary health care (PHC) systems (e.g. U.K., Australia, the Netherlands) explore barriers at three main levels: organizational, patient and provider level.

Previous research has indicated that the most common organizational barriers affecting maternal depression screening and management include: i) high work load and time restrictions, ii) absence of interdisciplinary team work, iii) lack of relevant health insurance coverage, iv) poor cooperation and collaboration between general and mental health sectors, v) inadequate community mental health services [29-31]. Facilitating factors include the establishment of supportive policies for family oriented healthcare and the integration of general health and mental health services [16,32]. The most frequently reported barriers at the pediatrician level include: i) reduced awareness and limited skills regarding basic aspects of maternal depression (clinical manifestation, impact, screening and management practices), ii) perceived roles and responsibilities, iii) poor perceived self-efficacy iv) fear of stigmatizing mothers, v) previous negative experiences in communicating with mental health professionals [33,34]. The importance of addressing the afore-mentioned obstacles at provider level is of high priority, since these barriers are common both in public and private pediatric settings and they can be more easily modified than the organizational ones [1,31,35,36].

Although there exist several surveys from countries with a strong primary health care system investigating the barriers to the early detection and management of maternal depression at the provider’s level, not many surveys exist from countries with weak primary health care systems. To our knowledge this is the first study conducted in a high income European country with a weak primary care system that investigates the primary care providers’ views on the detection and management of maternal depression. This is important, due to the negative impact of the weak primary health care system on the recognition and management of mental health problems and maternal depression [19,20,37]. Such an impact poses an additional risk for both mother and child.

The World Health Organization suggests that integration of mental health into primary care [38] is of high priority in order to address the mental health needs of the population, reduce the burden of mental diseases and promote the wellbeing and quality of life throughout the life course. Regrettably, in many high but also low and middle income countries (LMICs) [39] the appropriate changes to the primary healthcare system and the mental healthcare reform have not yet been established [40]. A weak primary care system is not necessarily a feature of a low or middle income country (LMIC). Many high-income countries such as Greece, Austria, Ireland and Iceland have a weak primary care system too. However, there is an increased possibility for LMICs to have a weak primary care system, due to the scarcity of resources and inequity of distribution [37].

Evidence shows [21,41,42] that both high income and LMICs with weak primary care systems are far from achieving the goal of integrating maternal mental health (and mental health in general) into routine primary care, mainly due to the following organizational obstacles: inadequate mental health expenditure, workforce deficits (e.g. lack of mental health specialists) and undertrained primary healthcare workers, insufficient decentralization of psychiatric services, inadequate or/and low quality community mental health services, limited or no collaboration between primary care and community settings and lack of public mental health leadership. Apart from the aforementioned organizational and systemic barriers, social exclusion, misconceptions about, and unfavorable attitudes towards maternal depression make its integration to primary care even more challenging [42].

The World Health Organization (2007) advocates that in the context of countries with weak primary healthcare systems the role of the health provider must be strengthened and upgraded for systemic barriers to be overcome, for accomplishing the integration of mental health into primary care and meet the mental health needs of the population. Primary healthcare providers should be trained and supervised appropriately to detect and refer individuals with mental health problems. In the present study we used a set of questions to explore the existing barriers at provider (pediatrician and health visitor) level to the recognition and management of maternal depression within the weak primary healthcare system in Greece.

## Methods

The present study is part of the research project “Building HealthCare Workers’ Capacity in Health Promotion: Development, Implementation and Evaluation of an Innovative Distance-Learning Intervention”. This is co-funded by the European Union (European Social Fund – ESF) and Greek national funds through the Program THALIS-University of Athens, of the Operational Program “Education and Lifelong Learning” of the National Strategic Reference Framework (NSRF). In its initial phase, the project aimed to assess the attitudes and skills of the healthcare professionals in Greece, and to identify obstacles and enablers, in order to guide the development, implementation and evaluation of an intervention *e-learning* program. One of the modules of the aforementioned project was “Maternal depression: Improving maternal mental health outcomes in the pediatric setting”. The present study intended to explore current practices as well as the perceptions of pediatricians and health visitors regarding the obstacles and facilitating factors in the detection and management of maternal depression.

The primary goal of the present study in terms of public health has been to inform policies and contribute to an improved involvement of the pediatric primary healthcare providers in the prevention of maternal depression. Our aim was not the generalizability of the results but the provision of critical information. Thus, we decided to use purposeful sampling because of our interest in, and focus on, “information rich” cases [43]. This information may be helpful to researchers, public health agents and policy makers, in shaping interventions and developing policies that concern the integration of maternal depression into the primary healthcare context.

Due to the financial circumstances in Greece, especially since 2012, it was deemed necessary to select participants who had been involved in the detection and management practices of maternal depression before 2012 and who continue to provide such services during the recession. In order to be eligible for the study, providers had to have at least five (5) years of professional experience and work at a primary pediatric setting as a pediatrician or health visitor. Seventy (70) pediatric primary health care providers (42 pediatricians and 28 health visitors) residing in Athens met the eligibility criteria. The financial crisis in Greece has escalated during the last two years leading to further budget cuts in health and social care. These changes may have differential influences on health professionals’ decisions regarding the provision of detection and management practices in cases of maternal depression but also the availability and acceptability of health and mental health services [44].

There is evidence that mental health services are more acceptable when they are decentralized and integrated into primary healthcare [19,20]. Moreover, research findings from previous studies indicated that Greeks display less favorable attitudes towards mental illness compared to people from other countries [26]. The outbreak of the financial crisis in Greece did not permit the completion of the mental health reform previously underway. Even though some reforms were implemented (e.g. the psychiatric beds in psychiatric hospitals were declined and the provision of psychiatric services was delegated to public general hospitals), a comprehensive mental health system was never developed due to budget cuts in healthcare as a response to the financial crisis. As a result, the mental health system remains underdeveloped. There exist few community mental health services, mainly in the large urban areas while most mental health needs are met by private-sector specialists (mainly psychiatrists).

There is evidence that in large urban areas the provision of care by community mental health services and educational campaigns have decreased the stigma attached to mental illness in the past [26]. Yet the recent weakening of community mental health services due to financial constraints and the provision of mental health mainly by specialists may have increased stigmatization and affected negatively the acceptability of mental health services. Consequently, we did not know whether community mental health services were considered (during the financial crisis) as acceptable by pediatric primary health providers who might have wanted to make a referral or for mothers who sought help. For this reason, we deemed necessary to include not just providers previously involved in detection and management practices, but those still providing such services, in order to ensure the collection of deeper and richer information hopefully leading to a comprehensive understanding to the matter at hand.

Each potential participant received an email with an invitation and instructions to contact a specific research team member to register for participation. Providers were selected to participate if they were involved in detection and management practices (identification and referral) of maternal depression in the last 12 months. Twenty two (22) pediatricians and eight (8) health visitors were excluded since they were not involved in detection and management practices in the previous 12 months, although they reported that in the past they had referred mothers for depression. Finally, 40 pediatric primary healthcare providers (20 pediatricians and 20 health visitors) were selected to participate. Twenty six (13 pediatricians and 13 health visitors) pediatric primary healthcare professionals agreed to participate and signed the informed consent document. The total sample included pediatric primary healthcare providers with different experience levels and from different regions in the Municipality of Athens. Health visitors and pediatricians from highly disadvantaged areas were also included in the study. Pediatricians recruited worked in pediatric primary healthcare settings while health visitors worked in Centers for Maternal and Child Protection.

Following the Institutional review board approval from the Medical School, University of Athens, the study was carried out in 2012–2013 in pediatric primary health care settings. The objectives of the study were:

• To investigate the awareness of pediatric primary healthcare professionals of maternal depression (impact, signs and symptoms etc.).

• To explore the current practices of pediatricians and health visitors in the identification and management (referral, counseling) of maternal depression.

• To investigate and describe the barriers to the detection and management of maternal depression as identified by pediatricians and health visitors.

• To explore and describe the facilitating factors in the recognition and management of maternal depression as identified by pediatricians and health visitors.

It was assumed that health visitors would display different opinions and patterns compared to pediatricians. In the Greek hospital-oriented primary care system, pediatricians (and physicians in general) enjoy a higher employment and power status compared to non-physician providers (e.g. health visitors). We decided to use interviews and not a different method (e.g. focus group discussions) for data collection in order to facilitate the free expression of providers’ opinions. Participants’ potential interplay in a focus group discussion might have resulted in a modification of their opinions.

An initial interview guide was developed, pilot tested and reviewed prior to the final administration. It contained a set of open-ended questions corresponding to the study objectives (Table 1). Face-to-face interviews, of approximately 45 minutes each, were conducted by experienced research team members in a participant’s preferred time and location. All interviews were audio-recorded and transcribed verbatim in Greek. At the beginning of each interview, participants answered questions about demographic characteristics (age, gender, specialty, setting and years of practice) (Table 2) and also an “ice-breaker” question, which was not analyzed.

**Table 1:** Interview questions

	**Mother’s mental health in general**
1	What do you think child mental health is? What do you think maternal mental health is?
2	How would you describe a mother of good mental health?
3	What kind of difficulties, if any, do you think mothers of young children (0–5) may be facing?
4	What kind of difficulties, if any, might a mother with depression be facing in an effort to seek help?
	**Signs and causes of depression**
5	How would you describe a mother experiencing depression?
6	What do you think about the main causes of depression in mothers of young children?
7	What worries you most about maternal depression?
8	What do you think are early signs of maternal depression?
9	How would you identify a mother with severe depression?
	**Dealing with maternal depression**
10	What do you think the reaction of the mother would be if you talked to her about depression?
11	If you suspect that a mother suffers from depression, what do you do?
12	What do you think about discussing depression with the mother of a patient?
	**Barriers and facilitators to detection and management of depression**
13	What barriers, if any, do you face in detecting mothers with depression or providing a referral for such mothers?
14	What would facilitate the detection and management of maternal depression in your setting?
15	What would increase your chances of implementing detection and management practices in the future?
16	Is there anything else you would like to mention?

**Table 2:** Participant demographics (n = 26)

	**n (%)**
**Gender**	
Male	7 (27)
Female	19 (73)
**Age** (years)	28–56 (range)
**Profession**	
Pediatricians	13 (50)
Health visitors	13 (50)
**Years practicing pediatric primary care**	
<5	8 (31)
5–10	12 (46)
>10	6 (23)
**Setting of practice**	
Public pediatric primary care centers	16 (62)
Private primary pediatric practice	10 (38)

The data were analyzed by using the framework analysis approach which includes five main steps: familiarization, identifying a thematic framework, indexing, charting, mapping and interpretation [45-48]. Three researchers were involved in the data elaboration and analysis with important contributions from the co-authors of this paper. First, the research team members read and re-read transcripts and listened to the recorded interviews to become familiar with the data set. Three research team members (a psychologist, a social researcher, a health policy researcher) coded the same four transcripts independently. Emerging themes and categories were used in developing a framework for coding and analyzing the data (by applying labels to the text chunks and passages in order to describe each notion in the text). All passages and chunks were coded the same way (the same label/code was given to the same concept/category). A series of formal meetings of the research team took place in order to develop and cultivate the thematic framework. The thematic framework was based on the *a priori* issues derived from the aims of the study as well as the concepts and themes that emerged from participants’ experiences. The research team members discussed the data thoroughly in order to agree (consensus) on an initial set of codes (each code with a brief definition) that would be applied to all transcripts. During the process of applying the codes to the remaining transcripts, systematic research team meetings were carried out to refine the analytical framework until no new codes emerged. A detailed index was articulated based on the recurring themes comprising the main and secondary categories and allowed for the refinement of the data. The line-by-line application of the thematic framework to all data resulted in a complete assignment of the data to main and secondary categories. At the end of this process, the data were sufficiently organized for analysis. Data was summarized by category (from each transcript), abstracted for each key theme and participant and inserted into the related matrix cell. The matrix included the refined summaries of the respondents’ experiences. The charting process provided researchers with a comprehensive view by theme and across cases. In the final stage of the framework analysis, charts were used to specify the main concepts but also to map and find associations among themes in the data so as to provide an interpretation for the results. The process of interpretation was carried out by taking into account the research objectives but also the emerging themes. We also searched for differences and common patterns in the views and experiences between and within groups (pediatricians and health visitors, public-private providers) and thematic areas. No differences between pediatricians and health visitors or between providers working in public and private sector were revealed across themes. Research team meetings were carried out to generate possible explanations of the phenomena emerging from the data.

## Results

Sample demographic characteristics are presented in Table 2. Most of the participants were female (73%) and practicing between 5–10 years (46%). It had been assumed that health visitors would hold different views and opinions compared to pediatricians. However, the analysis revealed that pediatricians and health visitors shared common views and practices and faced common barriers. We had also anticipated that providers working in public pediatric settings would point out different barriers compared to those working in private pediatric settings and hold different views. However, the analysis indicated that were no differences in opinions, self-reported barriers and practices between providers in the private and public sectors.

### Mother’s mental health in general

In the present study the meaning of “mental health” follows the definition provided by the World Health Organization [49] as “… a state of well-being in which the individual realizes his or her own abilities, can cope with the normal stresses of life, can work productively and fruitfully, and is able to make a contribution to his or her community”. The majority of the respondents provided descriptions of emotional wellbeing rather than mental health. They pointed to important aspects of maternal and child mental health such as the quality of mother-child interaction and relationship and woman’s adjustment to the role of motherhood.

*“The most important thing for child’s mental health is the establishment of a good relationship with the mother. Maternal mental health is a woman’s ability to adapt to motherhood and create a supportive environment for child development. Maternal mental health is dependent on achieving a balance between different roles”.* (HV 05)

*“A mentally healthy child is a happy child. The emotional state of a child is the most important thing for its mental health. And I have to say that child’s emotional state is easily observed by the pediatrician”.* (PED 11)

It should be noted that very few of the pediatric primary healthcare professionals acknowledged the importance of the antenatal and perinatal period to maternal mental health and also the fact that mental health is affected by several factors. Apart from mother’s appearance and childcare skills, many participants connected maternal mental health with the quality of communication and collaboration between mother, child and the pediatric health provider.

*“I guess that a mother with good mental health looks good, has a good relationship with her child… She is not anxious about the medical examination and as a result she can calm her child. She has an effective communication with the pediatrician and cooperates with the whole team very well”.* (PED 07)

All of the participants were aware of maternal depression and the importance of maternal mental health and wellbeing for the child’s development. They believed that it was part of their role to discuss mental health during well-child visits, although they stressed the importance of additional training in order to feel more confident.

*“It is definitely part of my role to detect depression and mental health problems in mothers, babies, toddlers and adolescents and provide referrals. But I need additional training. It’s not so simple… If I do not have appropriate training how can I provide appropriate help”?* (PED 01)

Participants acknowledged that mothers strive to meet the needs of their child and family. Moreover, they expressed increased concerns about the additional burden of the current financial crisis on maternal mental health.

*“If a mother continues to work after childbirth, she has a lot of problems… It is very difficult to combine motherhood and a job. It is too demanding. On the other hand, if a woman is unemployed, because this is [often] the case in Greece right now, she may feel that she has plenty of time with her baby, but she is not satisfied with her life and thus cannot manage everyday difficulties such as breastfeeding, the baby’s crying etc.… In our country, regulations for motherhood protection are insufficient or are not implemented -I don’t know*-”. (HV 13)

All health professionals mentioned that the lack of social and family support and also stigmatization are the main impediments preventing a mother from seeking help. They highlighted the lack of social and family support as either a cause or a consequence of stigmatization.

*“First of all, depression is stigmatized. If you are a mother you must be happy! You cannot say to your husband “you know, I want to give up all this, I can’t stand you or the child”. He will reject you and your feelings or he may underestimate the problem. In either case the mother will not receive support from husband and family. She may feel isolated and despair”.* (HV 10)

### Signs and causes of depression

Both types of providers relied mainly on observational signs to identify mothers with maternal depression. Withdrawal, sadness, inadequate response to baby’s cues, weight loss and frustration were the main signs mentioned. They described depressed mothers as overwhelmed, sad, displaying signs of neglect of personal and child hygiene, sustaining poor interaction with the provider and experiencing increased difficulty in following recommended preventive practices.

*“A mother with depression is usually very nervous and cannot deal with everyday issues such as her child’s feeding, dressing etc.… She gets mad or frustrated very easily…. She cannot recognize the child’s cry, needs and signals and she worries a lot about insignificant issues. There are also mothers who argue with me all the time and do not have a constant relationship with their child physician. They change pediatricians again and again, in an effort to seek help for themselves.* “(PED10)

*“She looks very tired and overwhelmed, she is sad. If you try to talk to her you will see that she cries very easily, she is very emotional. In many cases she is not interested in everyday activities and maybe life generally… You can see the emotional withdrawal… She looks so fragile…”* (HV 04)

It should be pointed out that participants found it hard to distinguish between early signs of depression and the clinical symptoms of a major depressive disorder. Only a few of them were able to make distinctions based on the frequency, intensity and duration of symptoms.

*“I cannot make such distinctions I am afraid…. Maybe a mother who is very demanding… Or the opposite, one who disregards important things. I think that an early sign of depression is when a mother reports exaggerated symptoms in relation to her child’s physical health without any reasonable grounds… Or [when] a mother is facing difficulties in following pediatric preventive practices or medication prescription. A mother with early depressive signs just tries to do whatever she can while a mother with major depression is emotionally withdrawn”.* (PED 05)

Participants attributed maternal depression mainly to the lack of the social and family support, financial problems and unemployment, while some mentioned a preexisting depression condition. Many of the health providers reported that the most severe consequences of maternal depression are self-harm or harm of others, abuse, neglect, and developmental problems for the child. Severe consequences associated with short-term outcomes were more easily pointed out by health providers, compared to medium depressive symptoms with long-term effects on maternal and child health.

*“First of all [there is] the possibility of infanticide or suicide, neglect, abuse. Apart from these, a mother with depression cannot meet sufficiently her child’s needs. She cannot support healthy choices in her child’s nutrition. Many children of depressive mothers eat unhealthy foods or they do not eat solid foods, as they should, according to their developmental stage”.* (PED 03)

*“Child abuse and neglect…The negative impact on a child’s mental and physical health. The failure to follow a vaccination schedule and preventive practices but also things such as… Infanticide, suicide or self-harming behaviors”.* (HV 01)

### Dealing with maternal depression

Most participants stated that they faced significant difficulties in discussing depression with mothers. They believed that mothers may be in denial or feel judged and stigmatized and would not be receptive to an option like referral. However, respondents reported that the establishment of rapport and an ongoing relationship with the mother might facilitate the maternal disclosure of depressive symptoms. They also pointed out that targeted training interventions are needed, focused on strategies about how to discuss depressive symptoms as opposed to a general training in communication skills. Furthermore, a majority of pediatric primary healthcare providers linked with stigmatization their personal difficulty in discussing depressive symptoms and implementing identification strategies. Significantly, they linked the avoidance of discussing mental health issues and maternal depression with stigmatization among pediatric primary healthcare providers.

*“It depends on my communication skills and the quality of the relationship which I have established with the mother up to that moment. I don’t know, I don’t feel very confident discussing with mothers about depression. I believe that they have a difficulty in talking about such issues. They may feel that I judge them, or that they are not good moms… I would like to find out how I can start a conversation with a mother about depression without making her feel uncomfortable. I need to know how to manage the situation”.* (HV 08)

*“I don’t feel very confident to talk about depression… Maybe I do not have the appropriate skills… But there is also stigma… I don’t know how to initiate such a conversation. A screening tool might help to start a discussion about depression. But what if the score indicates depression? What should I do? …I need a routine process for detecting and managing maternal depression. That would help”.* (PED 04)

As stated above, the providers relied mainly on observational signs to identify mothers with maternal depression. According to participants, the detection process of mothers with depression was comprised of two main steps. At first the pediatrician or health visitor looked for observable symptoms. In cases where such symptoms were perceived, then the health provider started asking questions to investigate the severity of the problem so as to decide whether it would be necessary to provide a referral. Asking questions is an essential but challenging process for most of the respondents. In terms of management practices, participants attempted to provide a referral for mild depression, but in cases of severe depression in addition to the referral they took action to ensure that both mother and infant were safe. Specifically, providers inform a woman’s husband/partner (or other close relatives in cases of divorce, death or absence of a husband/partner) about the situation and provide a referral in order to ensure that the mother would get help from a mental health specialist. In the next few days providers would contact the mother to ascertain that she received psychiatric care.

*“I try to discuss with the mother about her difficulties… It is very important to establish rapport and talk to her about the situation. I want to learn more about her thoughts, feelings, how she perceives the whole thing… After that I will provide a referral for a full psychiatric assessment”.* (HV 11)

*“In mild cases, I will try to suggest a visit to a mental health specialist for further help. I will try to talk about it in a way… you know… without talking about depression but talking about difficulties, let’s say… But if I believe that her child is in danger, I will be proactive. I will call her husband and family and provide referral immediately; I have to ensure that she and the child will be safe”.* (PED 02)

Many of the respondents believed that mothers were in denial and discussion about depression would increase stigmatization.

*“Not to mention that if I suggest a visit to a psychiatrist for example, she will answer,” Why are you suggesting that? Do you think I’m crazy?” and she will never talk to me again about depression.”* (HV 07)

*“Usually a mother is in denial. She does not suspect that she is facing problems with depression… I hesitate to suggest to her to visit a mental health specialist because of the stigma. I feel that if I say something like that, she will leave. On the other hand, if she looks for help and asks me about depression, I would feel more comfortable to discuss it. If she doesn’t… Well you know it may be difficult…”* (PED 08)

### Barriers and facilitators to detection and management of depression

#### *Barriers*

##### 

**Provider’s level** Health professionals in this study reported barriers at provider and systemic levels. All participants pointed out the lack of time and inadequate training as the main barriers in implementing detection and management practices in cases of maternal depression.

*“I do not have the time and readiness to respond always in an efficient way… Although I try to develop rapport and a supportive relationship with the mothers of my patients, it is difficult to achieve this with a depressive mother. I have never received such training”.* (PED 6)

*“I don’t feel very confident to discuss with a mother about depression. There is a lot of pressure at work, and I do not have the time. In many cases the system does not allow me to do my job. I mean they ask me to do the vaccination and stuff but do not pay attention to mental health issues. In my opinion mental health is not a priority for the Greek National Health System. Of course, if I had the training, maybe I ‘d be able to do something more… Maybe… It is very important for me to feel confident about my skills”.* (HV 12)

Apart from stigmatization, respondents mentioned that they have limited knowledge of the appropriate community referral resources. Although primary health providers stated that they tried to refer mothers to mental health specialists for additional help, their insufficient knowledge of community referral resources led to a decreased possibility for a mother of low socioeconomic status to get appropriate help.

*“Though we spend a lot of money as a country, we still don’t know the available community mental health services and we don’t use them”.* (HV 09)

Most pointed out that they were not aware of the available mental health services in their community and if they had to provide a referral, they would prefer a private mental health specialist.

*“I do not know the availability of community mental health services. As a result I would provide referrals to private mental health specialists and in many cases the patient cannot afford it”.* (PED 12)

*“There are important access difficulties to mental healthcare services. It is so unfair… There are community mental health services of good quality, but health professionals don’t know anything about them! As a result, many mothers do not receive the help they need because if we don’t know the public mental health services where we could refer them the only solution is a private mental health specialist. And the cost of this is usually high… I mean, how can you pay 40–50 euros per visit if you are unemployed?”* (HV 06)

Furthermore the participants believed that community mental health services were of low quality and availability. Additionally they emphasized the importance of training to address stigmatization since they reported that stigma is quite high among pediatric primary healthcare professionals.

*“Mothers in our country are suffering a lot until they find a solution. There is stigmatization especially for new mothers. Stigmatization does not only arise from friends and family but also the health professionals”.* (HV 06)

*“Motherhood in the context of the Greek society is considered as a state of happiness and fulfillment. A pediatrician, on the other hand, is a “good” physician who should confirm the above belief about motherhood and is not allowed to ask questions that may undermine it. I think that there is stigma in both cases… When I consider the problems mothers must face in order to find help for depression, I feel discouraged…Training is essential to address stigma and misconceptions about maternal depression”.* (PED 13)

### Systemic level

Most participants reported lack of free and available community mental health services, lack of an in-office mental health specialist, fragmentation of primary health services, lack of collaboration between primary health and mental health services and inadequate continuity of care as the core systemic barriers in the detection and management of maternal depression.

*“If I knew that there was an in-office mental health consultant or a mental health service in the community, free or at low cost, I would feel more comfortable to provide a referral”.* (PED 07)

*“A referral to a private mental health specialist poses an additional financial burden to the mother and decreases the possibilities of her receiving appropriate help”.* (HV 03)

*“There is no collaboration between health and mental health services, not to mention the weak and fragmented primary healthcare system… Continuity of care is not yet established in the public healthcare system”.* (PED 10)

### Facilitating factors

The majority of pediatric primary healthcare providers believed that training on maternal mental health issues and accurate screening questionnaires that are brief and easy to use in every day practice may significantly facilitate the detection process. The lack of confidence in being able to ascertain whether the observed symptoms are depressive makes the detection process even more challenging. Providers suggested that targeted training interventions will contribute to improving detection rates, by addressing stigma, increasing knowledge and developing skills that are essential to the identification and management of maternal depression.

*“I need a specific training in a standardized detection and management process… A step by step process that could be easily incorporated into my daily clinical practice. And also an easily accessed informational resource that I can trust”.* (PED 04)

*“If we had training, I think discussions would be easier, despite the additional barriers. I think training can overcome stigma to some extent…”* (HV 11)

Almost all health professionals believed that large-scale awareness campaigns could contribute to the elimination of depression stigmatization. They also suggested that targeted interventions for the prevention of maternal depression could increase both mother and family awareness and would result in a more favorable attitude of mothers regarding management practices.

*“TV spots and social media large-scale campaigns could increase awareness for stigmatization of depression and improve detection and mother’s family support”.* (HV 01)

Finally, many providers noted that the availability of a mental health specialist for consultation would result in improved detection rates as well as the improvement of collaboration between health and mental health services and the availability of low cost treatment options.

*“Of course, the collaboration between health and mental health professionals would help. If I could call a psychologist for example, and find out more about depression, I would feel more confident about my practices”.* (PED 03)

*“Quality community mental health services are essential but so is the collaboration of health and mental health professionals and services. A child may exhibit physical symptoms and developmental problems as a cause or consequence of maternal depression. We must have an effective way to refer [a patient] to an appropriate health or mental health service for additional help”.* (PED 06)

*“The availability of community mental health services at low cost. Easy access to mental health services without waiting for too long. To get the help you need at the right time”.* (HV 05)

## Discussion

Although participants in the present qualitative study reported that detection and management of maternal depression is part of their role, they stated that additional training is required in order for achieving the confidence to implement detection and management practices. Both pediatricians and health visitors acknowledged the importance of early detection and management of maternal depressive symptoms for preventing their negative impact on a child’s physical, mental and social health and development. On the other hand, health professionals identified barriers at many levels making them reluctant to discuss depression with mothers.

None of the pediatricians or health visitors who participated in the study reported the use of a screening tool in the assessment of depressive symptoms. Previous research indicated that relying on observation and other nonspecific methods is not an effective way to identify maternal depression [50] and most of the guidelines recommend the use of standardized screening tools in suspected cases of maternal and postpartum depression [51-53].

Important differences but also commonalities are revealed when comparing the present results with previous research findings. The main barriers found here have been highlighted in previous studies. The inadequate training on maternal mental health issues, the lack of time, the perceived low quality of community mental health services and the perception that mothers are in denial towards depressive symptoms, have been reported in previous qualitative studies [50,54,55].

The comparison of the present qualitative study carried out in a country with a weak primary health care system, with similar studies conducted in countries with strong primary care provides some interesting insights. In contrast to what Heneghan, Morton & DeLeone [50] have found, pediatricians and health visitors in our study believed that mothers were not receptive to discussing depression and accepting a referral from their child’s physician. Moreover, providers expressed concerns about initiating a conversation with mothers about depression and emphasized that they need specific training in order to feel confident enough to introduce the issue; otherwise their efforts to talk indirectly about depression are ineffective and doomed to fail. The majority of both pediatricians and health visitors acknowledged the fear of stigmatization as a key barrier in the detection and management of maternal depression and drew a connection between their hesitation to start a conversation about maternal depression and stigma. This differs from the findings of other studies. Olson and colleagues [56] have found that only 25% of pediatricians reported stigma as a barrier, and Heneghan, Morton & DeLeone [50] revealed that less than a third of pediatricians participating in their study have either reported stigma as a barrier or linked stigmatization as an obstacle to discussing the issue with mothers. Additionally, Chew-Graham and colleagues [55] suggested that health visitors did not cite fear of stigmatization as a barrier to talking to mothers about depression, however general practitioners did. Byat and colleagues [54] have also found that perinatal health professionals pointed to fear of stigmatization as a main obstacle for the recognition of maternal depression.

A possible explanation for the increased stigmatization reported among pediatric health professionals in the present study could be located in the context of the weak primary health care system. The result of the inefficiently decentralized psychiatric services and the provision of mental health services only by specialists at the secondary and tertiary health care level, is that maternal depression is considered as a specific health condition demanding pharmaceuticals and specialized care [41,42]. This enhances the stigmatization of, and misconceptions about, maternal depression and impedes the integration of maternal mental health into primary care [42]. In contrast, in countries with strong primary care, the stigmatization seems to be reduced mainly due to fact that primary health care services are not linked to a limited spectrum of health conditions and as such they are much more acceptable to, and accessible by, mothers and families [41,57]. Furthermore, the multi and inter disciplinary teamwork and the collaboration between community agencies and mental health services established within a strong primary health care system enable mothers and families to seek help and reduce social exclusion [40,42,58]. On the other hand, in countries with a weak primary care system cooperation between community health and mental health services is rare. In many cases the community organizations (e.g. nongovernmental organizations-NGOs) strive to establish their reputation and gain the trust of the community.

Another key difference between weak and strong primary healthcare systems is the approach of the healthcare providers in everyday practice. In general, providers in countries with weak primary care systems dominated by specialized services usually stick to the biomedical model [41]. They do not have adequate training in mental health issues and as a result they cannot adopt a holistic strategy in their daily routine, failing to implement patient-centered approaches [58]. Consequently, health providers working in a weak PHC system cannot achieve a sustained contribution to the increase of the mental health literacy of the population [59]. It should be noted that mental health literacy is associated with stigma, help-seeking behavior and recognition of mental health conditions [59].

Differences in the organization and staffing of pediatric primary healthcare settings across countries may have resulted in variations in findings across several qualitative studies. Heneghan, Morton & DeLeone [50] revealed that pediatricians in United States rely on social workers in order to address systemic barriers (e.g. lack of time) to the detection and management of maternal depression. Moreover in many countries (e.g. Canada, USA, U.K., Australia) pediatric and maternal primary healthcare is provided by multi-disciplinary healthcare teams or at least by a team consisting of a pediatrician and a health visitor or a registered nurse [55,60]. The limited resources in most pediatric primary healthcare settings in Greece make the incorporation of mental health issues in primary care more challenging. The provision of treatment almost exclusively by mental health specialists (e.g. psychiatrists), the providers’ perceived lack of expertise, and the perceived lack of services in primary healthcare settings may be the reasons why all health professionals in the present study considered referral as the only management option.

It was anticipated that pediatricians and health visitors would hold different views towards detection and management of maternal depression. The analysis revealed that they shared common patterns. This may suggest that the weak primary care system influences the providers of different professions in the same way and produces the same results. Within a weak primary care system, care is not provided by multi or interdisciplinary teams but by individual health professionals following a uni-disciplinary or para-disciplinary approach [24,61]. The absence of multidisciplinary teams in primary care is one of the core aspects of the weak primary care systems [24]. The pediatrician or the health visitor cooperates with the family and the patient, but does not collaborate with her/his colleagues [61]. As a result, providers face the same barriers towards maternal depression since there are not different duties or options regarding the identification and management of maternal depression. Both pediatricians and health visitors can implement the same detection practices and suggest the same management options. In addition they are equally undertrained regarding mental health issues.

It was also assumed that providers in the public sector would be holding different opinions from colleagues in the private sector. As stated in the introduction, the Greek health system comprises a combination of public, public contract and public reimbursement models and is based on public-private partnerships [26]. The patients have access to private pediatricians at no additional cost due to providers’ contractual agreements with social insurance funds. The care in the private pediatric settings in Greece is mainly provided by solo-practitioners [26]. This implies that the pediatrician in the private sector follows the same uni-disciplinary approach as the pediatrician in the public sector. This may be why they seemed to share opinions and face similar obstacles towards identification and management of maternal depression.

According to Lincoln & Guba [62] the findings of the present study are subjected to the following limitations. The study aimed to contextualize the detection and management of maternal depression within a weak primary healthcare system of a country in a financial crisis. Given this objective, the sample size should have been larger. It cannot be claimed that the aforementioned opinions expressed by health professionals are representative of the whole pediatricians’ and health visitors’ primary care population. Since the aim of the study was not the generalizability of the results but the provision of critical information, a purposeful sampling was used. This sampling method served the purpose of the study, but the generalizability of the results was limited. We decided to include pediatric primary care providers who were involved in preventive practices regarding maternal depression not only in previous years but also during the last 12 months. This implies that the sample of the present study has a special interest in maternal depression prevention. A comparison between professionals providing services only before the financial crisis and providers currently deliver detection and management services for maternal depression might have resulted in different findings.

The study was conducted in the context of a weak, hospital-oriented primary health care system outweighed (in terms of value and importance) by secondary and tertiary healthcare services [27]. Within this system, the staff and budget allocation is uniquely distributed in favor of the hospitals. As a result, primary care is weak and underdeveloped [26]. It was expected that within this system, pediatricians and health visitors would have different opinions and beliefs, due their different positions in the system’s hierarchy. In contrast to our hypothesis there were no differences in views and beliefs between pediatricians and health visitors towards detection and management of maternal depression. This may be attributed to the fact that the interviews were conducted with those who were willing or even more available to participate. Perhaps there were pediatricians and health visitors who were difficult to reach (e.g., due to workload) and whose opinions would differentiate the results.

As stated in the previous sections, the Greek primary health care system comprises a mixture of public, public contract and public reimbursement models, and is based on public-private partnerships. Thus, providers drawn from both the public and private sector were included in the study. On the other hand, all participants were residing in Athens. Athens is a large urban center and home to approximately 35% of the total Greek population [26]. Pediatric primary care providers in semi-rural and rural areas may have different opinions and views but were not included in the study. Thus the present findings reflect only the views of the pediatric primary care providers residing in Athens with a special interest in maternal depression prevention.

A common threat for credibility in qualitative studies is the social desirability bias [63]. Focus group discussions involve interaction between participants and therefore could have increased the social desirability bias, as both pediatricians and health visitors would try to present themselves as more responsible professionals and avoid express opinions about stigma (especially stigmatization among health professionals). We conducted face to face interviews and used experienced interviewers to create a non judgmental environment and facilitate providers to express their personal views freely. Furthermore we used probes to clarify the participants’ opinions and elicit detailed data, especially in cases of contradictory opinions and beliefs. Contradictory opinions and beliefs (e.g., participants stated that they do not make referral to community health services due to their limited information on the availability of such services but also because they believe that available services are of low quality) were not discarded but were included in detail in order to enhance transparency.

Data analysis was conducted by researchers from different backgrounds so as to increase its consistency and trustworthiness. Moreover, systematic debriefing sessions were carried out between the researchers responsible for analyzing the data. The researchers involved in data analysis had the opportunity to get feedback from the rest of the research team in an attempt to overcome possible prejudices. Regular research team meetings were also important for testing the interpretation of ideas and investigating alternative explanations for the phenomena, as an effort to enhance confirmability.

## Conclusions

Despite the limitations, the present study may provide useful information to understanding the factors associated with identification and management of maternal depression in countries with weak primary health care systems and limited resources. The present results suggest that specific aspects (e.g., fragmentation of health services, lack of collaboration between health and mental health services, provision of care only by specialists) of the weak primary health care system in Greece makes the detection and management of maternal depression more challenging. Therefore the role of the pediatric primary healthcare providers’ must be strengthened and upgraded for systemic barriers to be overcome, so as to achieve the integration of maternal depression into primary care and meet the mental health needs of the population.

## Competing interests

The authors declare that they have not competing interests.

## Authors’ contributions

EA: conceptualization, design, data analysis, preparation of manuscript, editing. KS: data analysis and elaboration, preparation of manuscript. SJ: design, comments on first and second draft, revising. VB: supervision of the data collection, editing. SC: comments on first draft, editing. CD: design, data analysis. YT: overall coordination, comments on first and second draft. All authors contributed to the interpretation of the data and have read and approved the final manuscript.

## Pre-publication history

The pre-publication history for this paper can be accessed here:

http://www.biomedcentral.com/1471-244X/14/108/prepub

## References

[B1] Leiferman JA Dauber SE Heisler K Paulson JF Primary care physicians’ beliefs and practices toward maternal depression J Women’s Health 2008 17 7 1143 1150 10.1089/jwh.2007.0543 18657043

[B2] Munk-Olsen T Laursen TM Pedersen CB Mors O Mortensen PB New parents and mental disorders: a population-based register study JAMA 2006 296 21 2582 2589 10.1001/jama.296.21.2582 17148723

[B3] Gaynes BN Gavin N Meltzer-Brody S Lohr KN Swinson T Gartlehner G Brody S Miller WC Perinatal depression: prevalence, screening accuracy, and screening outcomes Evid Rep Technol Assess (Full Rep) 2005 119 1 8 10.1037/e439372005-001 PMC4780910 15760246

[B4] Righetti-Veltema M Bousquet A Manzano J Impact of postpartum depressive symptoms on mother and her 18-month-old infant Eur Child Adoles Psychiatr 2003 12 2 75 83 10.1007/s00787-003-0311-9 12664271

[B5] Beck LF Morrow B Lipscomb LE Johnson CH Gaffield ME Rogers M Gilbert BC Prevalence of selected maternal behaviors and experiences, Pregnancy Risk Assessment Monitoring System (PRAMS), 1999 Morb Mortal Wkly Rep Surveill Summ 2002 51 2 1 27 12004983

[B6] Leonardou AA Zervas YM Papageorgiou CC Marks MN Tsartsara EC Antsaklis A Christodoulou GN Soldatos CR Validation of the Edinburgh postnatal depression scale and prevalence of postnatal depression at two months postpartum in a sample of Greek mothers J Reprod Infant Psyc 2009 27 1 28 39 10.1080/02646830802004909

[B7] Beardslee WR Bemporad J Keller MB Klerman GL Children of parents with major affective disorder: a review Am J Psychiatry 1983 140 7 825 832 6344660 10.1176/ajp.140.7.825

[B8] Beck CT Maternal depression and child behaviour problems: a meta-analysis J Adv Nurs 1999 29 3 623 629 10.1046/j.1365-2648.1999.00943.x 10210459

[B9] Mandl KD Tronick EZ Brennan TA Alpert HR Homer CJ Infant health care use and maternal depression Arch Pediatr Adolesc Med 1999 153 8 808 813 10.1001/archpedi.153.8.808 10437752

[B10] Minkovitz CS Strobino D Scharfstein D Hou W Miller T Mistry KB Swartz K Maternal depressive symptoms and children’s receipt of health care in the first 3 years of life Pediatrics 2005 115 2 306 314 10.1542/peds.2004-0341 15687437

[B11] Logan JE Riley AW Barker LE Parental mental and pain-related health and pediatric ambulatory care sensitive emergency department visits and hospitalizations Health Serv Res 2008 43 2 656 674 10.1111/j.1475-6773.2007.00790.x 18370972 PMC2442379

[B12] Flynn HA Davis M Marcus SM Cunningham R Blow FC Rates of maternal depression in pediatric emergency department and relationship to child service utilization Gen Hosp Psychiatry 2004 26 4 316 322 10.1016/j.genhosppsych.2004.03.009 15234828

[B13] Wertlieb D American Academy of Pediatrics Task Force on the F Converging trends in family research and pediatrics: recent findings for the American Academy of Pediatrics Task Force on the Family Pediatrics 2003 111 6 Pt 2 1572 1587 12777596

[B14] Sheeder J Kabir K Stafford B Screening for postpartum depression at well-child visits: is once enough during the first 6 months of life? Pediatrics 2009 123 6 e982 e988 10.1542/peds.2008-1160 19482749

[B15] Mishina H Takayama JI Screening for maternal depression in primary care pediatrics Curr Opin Pediatr 2009 21 6 789 793 10.1097/MOP.0b013e328331e798 19726991

[B16] Heneghan AM Chaudron LH Storfer-Isser A Park ER Kelleher KJ Stein RE Hoagwood KE O’Connor KG Horwitz SM Factors associated with identification and management of maternal depression by pediatricians Pediatrics 2007 119 3 444 454 10.1542/peds.2006-0765 17332196

[B17] Thompson C Kinmonth AL Stevens L Peveler RC Stevens A Ostler KJ Pickering RM Baker NG Henson A Preece J Cooper D Campbell MJ Effects of a clinical-practice guideline and practice-based education on detection and outcome of depression in primary care: Hampshire Depression Project randomised controlled trial Lancet 2000 355 9199 185 191 10.1016/S0140-6736(99)03171-2 10675118

[B18] Jane-Llopis E Anderson P Mental Health Promotion and Mental Disorder Prevention 2005 A policy for Europe. In. Nijmegen: Radboud University Nijmegen

[B19] Wittchen HU Muhlig S Beesdo K Mental disorders in primary care Dialogues Clin Neurosci 2003 5 2 115 128 22034245 10.31887/DCNS.2003.5.2/huwittchen PMC3181625

[B20] Ngo VK Rubinstein A Ganju V Kanellis P Loza N Rabadan-Diehl C Daar AS Grand challenges: Integrating mental health care into the non-communicable disease agenda PLoS Med 2013 10 5 e1001443 10.1371/journal.pmed.1001443 23690753 PMC3653779

[B21] Jacob KS Sharan P Mirza I Garrido-Cumbrera M Seedat S Mari JJ Sreenivas V Saxena S Mental health systems in countries: where are we now? Lancet 2007 370 9592 1061 1077 10.1016/S0140-6736(07)61241-0 17804052

[B22] Kringos S The Strength of Primary Care in Europe 2012 NIVEL: The Netherlands

[B23] Starfield B Shi L Macinko J Contribution of primary care to health systems and health Milbank Q 2005 83 3 457 502 10.1111/j.1468-0009.2005.00409.x 16202000 PMC2690145

[B24] Shi L The impact of primary care: a focused review Scientifica (Cairo) 2012 2012 432892 24278694 10.6064/2012/432892 PMC3820521

[B25] Macinko J Starfield B Erinosho T The impact of primary healthcare on population health in low- and middle-income countries J Ambul Care Manage 2009 32 2 150 171 10.1097/JAC.0b013e3181994221 19305227

[B26] Economou C Greece: Health system review Health Syst Transit 2010 12 1 177 21330233

[B27] Tountas Y Karnaki P Pavi E Reforming the reform: the Greek National Health System in transition Health Pol 2002 62 1 15 29 10.1016/S0168-8510(01)00217-2 12151132

[B28] Simou E Karamagioli E Roumeliotou A Reinventing primary health care in the Greece of austerity: the role of health-care workers Prim Health Care Res Dev 2013 1 9 Epub ahead of print 24252634 10.1017/S1463423613000431

[B29] Horwitz SM Kelleher KJ Stein RE Storfer-Isser A Youngstrom EA Park ER Heneghan AM Jensen PS O’Connor KG Hoagwood KE Barriers to the identification and management of psychosocial issues in children and maternal depression Pediatrics 2007 119 1 e208 e218 10.1542/peds.2005-1997 17200245

[B30] Lofrumento M Stone S, Menken A The Pediatrician’s Role in Identifying Postpartum Mood Disorders Perinatal and Postpartum Mood Disorders 2008 New York: Springer Publishing Company

[B31] Wiley CC Burke GS Gill PA Law NE Pediatricians’ views of postpartum depression: a self-administered survey Arch Women’s Mental Health 2004 7 4 231 236 10.1007/s00737-004-0058-4 15480860

[B32] Earls MF Hay SS Setting the stage for success: implementation of developmental and behavioral screening and surveillance in primary care practice–the North Carolina Assuring Better Child Health and Development (ABCD) Project Pediatrics 2006 118 1 e183 e188 10.1542/peds.2006-0475 16818532

[B33] Currie ML Rademacher R The pediatrician’s role in recognizing and intervening in postpartum depression Pediatr Clin N Am 2004 51 3 785 801 xi 10.1016/j.pcl.2004.01.008 15157598

[B34] Gjerdingen DK Yawn BP Postpartum depression screening: importance, methods, barriers, and recommendations for practice J Am Board Fam Med 2007 20 3 280 288 10.3122/jabfm.2007.03.060171 17478661

[B35] Chaudron LH Szilagyi PG Kitzman HJ Wadkins HI Conwell Y Detection of postpartum depressive symptoms by screening at well-child visits Pediatrics 2004 113 3 Pt 1 551 558 14993549 10.1542/peds.113.3.551

[B36] Olson AL Dietrich AJ Prazar G Hurley J Brief maternal depression screening at well-child visits Pediatrics 2006 118 1 207 216 10.1542/peds.2005-2346 16818567

[B37] Saxena S Thornicroft G Knapp M Whiteford H Resources for mental health: scarcity, inequity, and inefficiency Lancet 2007 370 9590 878 889 10.1016/S0140-6736(07)61239-2 17804062

[B38] WHO World Health Report 2001: Mental Health: new Understanding, New Hope 2001 Geneva: World Health Organization

[B39] Bank W 2013 World Development Indicators 2013 Washington DC: The World Bank

[B40] Collins PY Insel TR Chockalingam A Daar A Maddox YT Grand challenges in global mental health: integration in research, policy, and practice PLoS Med 2013 10 4 e1001434 10.1371/journal.pmed.1001434 23637578 PMC3640093

[B41] WHO Expert Opinion on Barriers and Facilitating Factors for the Implementation of Existing Mental Health Knowledge in Mental Health Services 2007 Geneva: Department of Mental Health and Substance Abuse. World Health Organization

[B42] Rahman A Surkan PJ Cayetano CE Rwagatare P Dickson KE Grand challenges: integrating maternal mental health into maternal and child health programmes PLoS Med 2013 10 5 e1001442 10.1371/journal.pmed.1001442 23667345 PMC3646722

[B43] Devers KJ Frankel RM Study design in qualitative research–2: Sampling and data collection strategies Education Health 2000 13 2 263 271 10.1080/13576280050074543 14742088

[B44] Pappa E Kontodimopoulos N Papadopoulos A Tountas Y Niakas D Investigating unmet health needs in primary health care services in a representative sample of the Greek population Int J Environ Res Public Health 2013 10 5 2017 2027 10.3390/ijerph10052017 23685827 PMC3709361

[B45] Richie JSL Burgess B Qualitative Data Analysis for Applied Policy Research Analysing Qualitative Data 1994 London: Routledge 173 194

[B46] Pope C Ziebland S Mays N Qualitative research in health care. Analysing qualitative data BMJ 2000 320 7227 114 116 10.1136/bmj.320.7227.114 10625273 PMC1117368

[B47] Srivastava P Hopwood N A practical iterative framework for qualitative data analysis Int J Qual Meth 2009 8 1 76 84

[B48] Lacey A Luff D Trent Focus for Research and Development in Primary Health Care: An Introduction to Qualitative Analysis Trent Focus 2001 Vancouver, Canada: University of British Columbia

[B49] WHO Investing in Mental Health: Evidence for Action 2013 2001 Geneva: Switzerland: World Health Organization

[B50] Heneghan AM Morton S DeLeone NL Paediatricians’ attitudes about discussing maternal depression during a paediatric primary care visit Child Care Health Dev 2007 33 3 333 339 10.1111/j.1365-2214.2006.00648.x 17439448

[B51] Calonge N Petitti DB DeWitt TG Dietrich AJ Gordis L Gregory KD Harris R Isham G LeFevre ML Leipzig RM Loveland-Cherry C Marion L Moyer V Ockene J Sawaya G Yawn B Screening for depression in adults: U.S. Preventive Services Task Force recommendation statement Ann Intern Med 2009 151 11 784 792 19949144 10.7326/0003-4819-151-11-200912010-00006

[B52] Hagan J Shaw JS Duncan P (Eds) Bright futures guidelines for health supervision of infants, children, and adolescents Pocket Guide 2008 3 Elk Grove Village, IL: American Academy of Pediatrics

[B53] NICE Antenatal and Postnatal Mental Health. National Costing report. Implementing NICE Guidance in England 2007 London: National Institute for Health and Clinical Excellence

[B54] Byatt N Biebel K Lundquist RS Moore Simas TA Debordes-Jackson G Allison J Ziedonis D Patient, provider, and system-level barriers and facilitators to addressing perinatal depression J Reprod Infant Psyc 2012 30 5 436 449 10.1080/02646838.2012.743000

[B55] Chew-Graham CA Sharp D Chamberlain E Folkes L Turner KM Disclosure of symptoms of postnatal depression, the perspectives of health professionals and women: a qualitative study BMC Fam Pract 2009 10 7 10.1186/1471-2296-10-7 19159478 PMC2637839

[B56] Olson AL Kemper KJ Kelleher KJ Hammond CS Zuckerman BS Dietrich AJ Primary care pediatricians’ roles and perceived responsibilities in the identification and management of maternal depression Pediatrics 2002 110 6 1169 1176 10.1542/peds.110.6.1169 12456915

[B57] WHO What is primary care mental health?: WHO and Wonca Working Party on Mental Health Ment Health Fam Med 2008 5 1 9 13 22477841 PMC2777553

[B58] Patel V Belkin GS Chockalingam A Cooper J Saxena S Unutzer J Grand challenges: integrating mental health services into priority health care platforms PLoS Med 2013 10 5 e1001448 10.1371/journal.pmed.1001448 23737736 PMC3666874

[B59] Jorm AF Mental health literacy. Public knowledge and beliefs about mental disorders Br J Psychiatry 2000 177 396 401 10.1192/bjp.177.5.396 11059991

[B60] Pringle D Levitt C Horsburgh ME Wilson R Whittaker MK Interdisciplinary collaboration and primary health care reform Statement from the Ontario Chairs of Family Medicine and the Council of Ontario University Programs in Nursing Can Fam Physician 2000 46 76 765 771-764 PMC2144836 10790804

[B61] Satin G Satin G The Interdisciplinary, Integrated Approach to Professional Practice with the Aged The Clinical Care of the Aged Person: An Interdisciplinary Perspective 1994 New York: Oxford University Press 391 403

[B62] Lincoln Y Guba E Naturalistic Inquiry 1985 Canada: Sage Publications

[B63] Krefting L Rigor in qualitative research: the assessment of trustworthiness Am J Occup Ther 1991 45 3 214 222 10.5014/ajot.45.3.214 2031523

